# Outcome of complicated osteoarticular brucellosis in a tertiary care center in Saudi Arabia

**DOI:** 10.1371/journal.pone.0299878

**Published:** 2024-03-27

**Authors:** Ebrahim Mahmoud, Areej Alaman, Raghad Alsayari, Anadel Hakeem, Mohammad Bosaeed, Azaheer Ibrahim, Saleh Algazlan, Abdullah Almanea, Ahmed A. Abulaban

**Affiliations:** 1 Division of Infectious Diseases, Department of Medicine, King Faisal Specialist Hospital and Research Center, Madinah, Saudi Arabia; 2 Department of Medicine, King Abdulaziz Medical City, Ministry of National Guard-Health Affairs, Riyadh, Saudi Arabia; 3 King Abdullah International Medical Research Center, Riyadh, Kingdom of Saudi Arabia; 4 College of Medicine, King Saud bin Abdulaziz University for Health Sciences, Riyadh, Kingdom of Saudi Arabia; 5 Division of Neurology, King Abdulaziz Medical City, Ministry of National Guard-Health Affairs, Riyadh, Saudi Arabia; Bangladesh Agricultural University, BANGLADESH

## Abstract

**Objective:**

To evaluate the outcome of complicated osteoarticular brucellosis.

**Methods:**

A retrospective chart review was conducted at King Abdulaziz Medical City (KAMC), in Riyadh, Saudi Arabia. All patients aged more than 14 who have been diagnosed with complicated brucellosis with osteoarticular disease between July 2016 and December 2022 were included.

**Results:**

A total of 82 (10.7%) patients met the criteria, with a male predominance of 66 (80.4%), and their mean age was 56.4 ± 19.3 years. A positive blood culture was found in 33 (40.2%). The most common clinical presentation was fever (57.3%). All patients received a doxycycline-based regimen except one. 62 (75.60%) patients were treated with three or more medication regimens, while 20 (24.40%) patients received two drug regimens. The mean duration of therapy was 94.2 days for two-drug therapy and 116.4 days for three-drug therapy. A total of 78 out of 82 (95.1%) cases were cured at the end of treatment. Unfavorable outcomes were documented in four cases (two relapses and two treatment failures). Neither using three drugs regimen nor longer duration of therapy was associated with better outcome.

**Conclusions:**

Unfavorable outcomes have been noticed to be minimal in our cohort of patients with osteoarticular brucellosis, treated mainly with a three-drug regimen and a longer duration of therapy.

## Introduction

Brucellosis is the most common zoonotic disease [1–3]. More than 500,000 new cases have been reported annually in the world, with the majority of the cases in developing regions where the disease is endemic [4]. In Saudi Arabia, 3661 *Brucella* infection cases were reported in 2021 [5].

Brucellosis disease is caused by gram-negative, non-capsulated coccobacilli [6]. It spreads by consuming raw dairy products from infected animals, meat, and close contact with their bodily fluids [7]. The disease usually causes a non-localized illness that manifests as fever, profuse sweating, general malaise, low backache, and musculoskeletal pain. However, the infection may affect specific body organs and cause localized diseases like septic arthritis, spondylitis, spondylodiscitis, endocarditis, orchitis, and other complications, including neurologic disease [8, 9]. Nevertheless, osteoarticular involvement is the most common complication of brucellosis, with a reported incidence of 10–85% in most series [10–13].

Despite this large number of cases and the endemicity of brucellosis in our region; there is still limited data regarding the outcome of complicated brucellosis, namely osteoarticular disease. While most of the evidence focuses more on the prevalence of the disease [7, 14, 15], we aimed to describe the clinical outcome associated with localized brucellosis in the bone and joint.

## Material and method

### Study design

This is a retrospective chart review study conducted at King Abdulaziz Medical City (KAMC), which is a tertiary care center in Riyadh, Saudi Arabia, with a bed capacity of over 1500 and provides care to a diverse patient population with complex diseases.

### Study population and definitions

Patients who were more than 14 years old and had been diagnosed with complicated brucellosis with osteoarticular disease between July 2016 and December 2022 were included. Their data was accessed and reviewed between 28^th^ October 20222 and 31^st^ of May 2023.

Data collected from the patient records included demographics, clinical data in terms of clinical history, osteoarticular involvement, investigations, microbiologic data, radiological imaging, type and duration of antibiotics prescribed, mortality, as well as follow-up for the patient and the assessment of the response to therapy.

Patients who were previously treated for brucellosis were excluded.

The slide agglutination test (Quimica Clinica Aplicada, QCA) was used as a semiquantitative determination for both *Brucella abortus* and *melitensis* titers. *Brucella* culture was isolated using an automated system (BD BACTEC™ Automated System, from Becton, Dickinson and Company) that also provided antimicrobial susceptibility testing results.

#### Definition

*Complicated osteoarticular brucellosis*. Diagnosis of brucellosis based on positive serology equal to or more than 1:320 and/or positive blood culture with osteoarticular disease.

Osteoarticular involvement was defined as bone or joint involvement clinically (based on the primary physician’s evaluation and discretion) or radiologically as evidence of bone, spine, or joint involvement at the same time or after brucellosis diagnosis or a positive bone or joint culture of brucellosis.

*Cure*. The assessment of cure was based on a chart review and the treating physician’s evaluation of whether the patient’s initial complaint was improving or not. The cure was classified as

1. Only a clinical cure based on the clinical presentation (resolution of all symptoms including fever, musculoskeletal pain and other complains patient presented with).
2. Clinical and laboratory: clinical response and reduction of inflammatory markers by more than 80% or normalized inflammatory markers.
3. Radiology: resolution of at least 90% of the initial lesions in the follow-up imaging, if the imaging was done.

*Relapse*. Reoccurrence of symptoms (without other explanation) and/or a positive blood culture after the treatment was concluded within one year of follow-up.

Also, persistence of the symptoms while the patient was on therapy for more than 2 weeks was included and labeled as treatment failure.

*Treatment*. Any medication lasting more than two weeks is considered, except aminoglycoside medication if it lasts more than five days.

*Mortality*. Died within 90 days from the time of starting therapy.

### Objective/Outcome

The primary outcome of this study was to evaluate the outcome (cure rate and relapse) of the complicated brucellosis at the end of therapy. Patients were later sub-grouped based on whether they received treatment for more than 3 months or less than 3 months. As well as whether they received two medications or three medications during the treatment.

Secondary objectives included comparing the different regimens and durations used for the treatment. Also, to determine the distribution and clinical characteristics of the disease.

### Statistical analysis

We reported categorical data as frequencies and percentages, while continuous ones were reported as mean ± standard deviation. Univariable analysis was done. T-test and Chi square were used where appropriate. Multivariable analysis was not performed due to small sample size and rare outcome. All tests were considered statistically significant if the p-value was less than 0.05. The statistical analysis was done by The Statistical Package for the Social Sciences (SPSS), version 22 (IBM Corp., Armonk, NY, USA).

### Ethical statement

The study was approved by the Institutional Review Board (IRB) at King Abdullah International Medical Research Center (KAIMRC; protocol number: NRC22R /368/08). Written informed consent was waived by the IRB as the study was a retrospective chart review, where research involves no more than minimal risk to the subjects. The study complied with the Declaration of Helsinki concerning maintaining the confidentiality of the patient’s data as the data were anonymized. The data was accessed and reviewed between 28^th^ October 20222 and 31^st^ of May 2023.

## Result

### Study population

Out of 765 patients with positive brucella serology between 2016 and 2022, a total of 82 (10.72%) met the definition of complicated brucellosis. The patients were predominantly male (66 patients, 80.4%), and their mean age was 56.4 ± 19.3 years. Further baseline characteristics are described in (Table 1).

**Table 1:** Baseline demographic characteristics of complicated brucellosis.

	*Total 82*	*Triple therapy*x*	*Dual therapy*
	*n = (%)*	*(62 patients)*	*(20 patients)*
Age, y (median) **	60 median, 56.4 ± 19.3	56.3 ± 19.3	56.3 ±20.4
Gender; Male	66 (80.5)	51 (22.7)	15 (77.3)
Duration of therapy**	111± 61.4	116.4 ±65.8	94.2 ±44.6
*Comorbidities*			
Congestive heart failure	6 (7.3)	4 (66.7)	2 (33.3)
Renal disease, Estimated Glomerular Filtration Rate (eGFR) < 60 ml/min	1 (1.2)	1 (100)	0 (0)
Hypertension	32 (39.0)	26 (81.3)	6 (18.8)
Diabetes mellites	35 (42.7)	26 (74.3)	9 (25.7)
*Diagnosis*			
Blood culture positive	33 (40.2)	27 (81.8)	6 (18.2)
Positive culture from sample	12 (14.6)	10 (83.3)	2 (16.7)
Radiological finding	66 (80.5)	53 (80.3)	13 (19.7)
*Pattern of involvement*			
Spine	55 (67.1)	40(72.7)	15 (27.3)
Joint	23 (28.0)	19 (82.6)	4 (17.4)
Bone	4 (4.9)	3 (75.0)	1 (25.0)
Baseline labs**			
WBC (109/L)	8.9 ±2.2	7.2 ± 2.0	6.1 ±2.4
Hemoglobin (g/L)	129.2 ± 21.3	127.8 ±16.5	128.6± 18.5
Platelet count (109/L)	293.9± 87.5	296.6± 86.69	285.2 ± 93.9
ESR (***mm/hr***)	64.2 ± 32.3	65.4 ±29.3	59.7 ±43.2
CRP (mg/L)	64.0 ± 59.1	66.7 ±62.1	49.2 ±47.3
*Symptoms*			
Fever	47 (57.3)	31 (66.0)	16 (34.0)
Night sweat	22 (26.8)	15 (68.2)	7 (31.8)
Weight loss	5 (6.1)	1 (20.0)	4 (80.0)
Arthralgia	31 (37.8)	24 (77.4)	7 (22.6)
Back pain	29 (35.4)	21 (72.4)	8 (27.6)
Hip pain	8 (9.7)	7 (87.5)	1 (12.5)
Knee pain	10 (12.2)	8 (80.0)	2 (20.0)
Fatigue	4 (4.9)	3 (75.0)	1 (25.0)
Ankle pain	5 (6.1)	2 (40.0)	3 (60.0)
Chills	4 (4.9)	3 (75.0)	1 (25.0)
Generalized lymphadenopathy	1 (1.2)	0(0)	1(100.0)
*Surgery*	16 (19.5)	15 (93.8)	1 (6.3)
*Outcome*			
Relapse	2 (2.4)	2 (100)	0 (0)
Failure	2 (2.4)	1 (50.0)	1 (50.0)
Died within 90 days	1 (1.2)	1 (100)	0 (0)

* One patient received 4 medications.

** T- test has been used

The median for *Brucella abortus* titers was 1280 and 1920 for *Brucella melitensis*. A positive blood culture was found in 33 (40.2%) patients, while 12 (14.6%) cultures grew Brucella from joint or bone tissue or fluid.

### Clinical symptoms, signs, and laboratory test results

The most common clinical initial presentation was fever in 57.3% of cases, followed by joint pain and back pain in 37.8% and 35.4%, respectively. More details are in (Table 1).

The pattern of musculoskeletal involvement was as follows: spine in 55 cases (32 spondylodiscitis, 16 sacroiliitis), joint in 23 cases (15 involved knee, 6 hip), and bone in 4 cases (2 femur).

Radiological evidence of involvement was documented by MRI in 66 (80.5%) patients: 38 (57.6%) cases of spondylodiscitis or discitis—most commonly involving the lumbar segment; 33 (86.8%)—which was associated with epidural/paraspinal abscess or phlegmon in 15 (22.7%) cases; and central canal stenosis in seven cases (six at the lumbar segment and one at the thoracic segment). Only one case (1.5%) of cord compression was encountered in association with lumbar spondylodiscitis and central canal stenosis. Nevertheless, all the cases diagnosed with different involvement (Spine, joint, bone) were supported by radiological imaging.

High inflammatory markers were the most common lab abnormality: 66 (80.4%) patients had an ESR greater than 30, and 64 (78%) patients had a CRP greater than 6.

A total of 16 (19.5%) patients underwent surgery, ten of whom were spine cases (six patients underwent decompression of the spine and four spine instrumentation), five patients underwent knee debridement and irrigation, and one patient underwent hip replacement.

### Outcome

A total of 78 out of 82 (95.1%) cases were cured at the end of treatment, at least clinically. Cure was classified as follows: 33 (40.24%) by clinical judgment, 28 (34.14%) by clinical and lab, and 12 (14.63%) by clinical, lab, and radiological. In five patients, the cure was judged to be clinical and radiological.

Unfavorable outcomes were documented in four cases. Two (2.4%) cases of relapse (one on a three-drug regimen and one on a four-drug regimen) and two cases of clinical failure (one on a two-drug regimen and one on a three-drug regimen). Three of the patients with unfavorable outcomes received treatment for less than three months, while one patient developed a relapse after six months of treatment.

Although some factors were associated with unfavorable outcomes (weight loss and lower limb weakness) but the number of samples was too small to qualify for statistical significance (Table 2). Multivariable analysis was not performed due to the small sample size and rare outcome noticed.

**Table 2:** Result of Univariable analysis of baseline factors of unfavourable outcome (relapse or failure at end of therapy)*.

	*Cure rate*	*Bad outcome (death*, *failure*, *and relapse)*, *n/N (%)*	Odd ratio (Confidence Interval)	P value
	*n/N (%)*			
Gender, male	63/66 (95.5%)	3/66 (4.5%)	0.33 (0.05–2.18)	0.23
*Diagnosis*				
Radiological	62/66 (93.9%)	4/66 (6.1%)	0.96(0.10–9.29)	0.97
*Symptoms*				
Fever	44/47 (93.6%)	3/47 (6.4%)	1.12 (0.17–7.12)	0.90
Weight loss	3/5 (60.0%)	2/5 (40.0%)	16.44 (1.95–138.27)	**0.001**
Back pain	26/29 (89.7%)	3/29 (10.3%)	2.94 (0.46–18.72)	0.23
Lower limb weakness	1/2(50.0%)	1/2 (50.0%)	19.00 (0.99–362.48)	0.009
Sweating	19/22 (86.4%)	3/22 (13.6%)	4.57 (0.71–29.49)	0.08
***Surgery***	15/16 (93.8%)	1/16 (6.3%)	1.03 (0.10–9.92)	0.97
***3 drug regimens***	58/62 (93.5%)	4/62 (6.5%)	1.31 (0.13–12.45)	0.81

* Chi-square tests has been used in the univariate analysis.

### Regimen used

All patients received a doxycycline-based regimen except one patient due to medication intolerance, who received a combination of rifampicin, ciprofloxacin, and streptomycin (RCS).

A total of 62 (75.60%) patients were treated using three or more medication regimens, while 20 (24.40%) patients received two-drug regimens as follows: six (7.3%) received doxycycline with streptomycin (DS), four (4.9%) received doxycycline with ciprofloxacin (DC), and ten (12.2%) received doxycycline with rifampin (DR). The mean duration of therapy was 94.2 days for two-drug therapy and 116.4 days for three-drug therapy.

Concerning the three-drug regimen, around half of the patients received either doxycycline, ciprofloxacin, and streptomycin (DCS) (29 or 35.4%) or doxycycline, rifampin, and streptomycin (DRS) (19 or 23.2%). More details about the regimen used (Table 3).

**Table 3:** Outcome therapy with different regimen in 82 patients; Triple therapy 62, (75.60%) and Dual therapy20, (24.40%).

Regimen of therapy (No. of cases)	Duration for 3 months or less N (%)	Duration for > 3 months N (%)	Relapse N (%) or Failure N(%)	Cure rate N (%)
**Triple therapy 62,(75.60%)**				
DCS (29)	16 (55.2%)	13 (44.8)	1 (3.4%) ^C^	28 (96.6%)
DRS (19)	15 (78.9%)	4 (21.1%)	1 (5.3%) ^B^	18 (94.8%)
RCS (1)	1 (100%)	0 (0)	0 (0)	1 (100%)
DRG (3)	2 (66.6%)	1 (33.4%)	0 (0)	3 (100%)
DBS (2)	2 (100%)	0 (0)	0 (0)	2 (100%)
DCBG (2)	1 (50%)	1 (50%)	1 (50%) ^a^	1 (50%)
DCR (1)	0 (0)	1 (100%)	0 (0)	1 (100%)
DCRS (1)	0 (0)	1 (100%)	0 (0)	1 (100%)
DCG (3)	3 (100%)	0 (0)	0 (0)	3 (100%)
DRCG (1)	1 (100%)	0 (0)	1 (100%) ^C^	0 (0)
***Dual therapy20*, *(24*.*40%)***				
DS (6)	6 (100%)	0 (0)	0 (0)	6 (100%)
DC (4)	3 (75%)	1 (25%)	0 (0)	4 (100%)
DR (10)	5 (50.0%)	5 (50.0%)	1 (10.0%) ^B^	9 (90.0%)

D: doxycycline, C: ciprofloxacin, R: rifampin, G: gentamycin, S: Streptomycin, B: Bactrim

^a^: died

^B^: failed treatment

^C^: relapse.

Only five (6.1%) patients had side effects from the medication, and four of them led to the discontinuation of therapy. Three had side effects related to ciprofloxacin (QT prolongation, neutropenia, and allergy type-1 hypersensitivity); two GI upsets occurred, one related to doxycycline and the other to rifampicin.

### Failure/Relapse cases

#### Failure

The first case was a 76-year-old male who presented with back pain and fever with weight loss and was diagnosed with brucellosis L5/S1 discitis with a Brucella titer of 1:320. He underwent abscess drainage and was treated with a two-drug regimen (doxycycline and rifampin) for 45 days, but his back pain did not improve. He was treated by another hospital with the same course for another 45 days with no improvement. So, he was started on a three-drug regimen (streptomycin with doxycycline and ciprofloxacin) for a total of 4 months. The patient presented to the clinic 2 months after that with 90% improvement in his back pain and also improvement in inflammatory markers and Brucella titer.

The second case was a 74-year-old male presented with back pain, fever, and night sweats that were initially misdiagnosed as non-localized brucellosis with Brucella titer 1:2560 and started on three-drug therapy (doxycycline, rifampin, and streptomycin). After 21 days, fever and night sweat improved, but back pain did not improve, and the titer increased to 1:5120 for that spine MRI was done and showed L2/L3 spondylodiscitis with prevertebral and paraspinal muscle involvement. The patient received treatment for two months initially, missed the follow-up and returned for follow-up again after one month. The patient then received treatment again for another three months, and he followed up after two months from treatment with a titer of 1:160 and an ESR normalized to 54.

#### Relapses

The third case was a 77-year-old male was admitted with a case of lower back pain for 8 months with radiculopathy that progressed until he became bedridden. He presented with spondylodiscitis with multilevel involvement at T8, T9, L3, L4, L5, and S1. The Brucella titer was 1:5120, and a CT-guided biopsy grew Brucella. Due to the severity of the disease, a four-drug regimen (rifampin, doxycycline, gentamicin, and ciprofloxacin) was followed. The patient received treatment for 60 days, stopped medications by himself due to nausea and vomiting and was admitted again after 2 months. He was again started on rifampin, doxycycline, and gentamicin with good tolerance for 3 months with good clinical response but with no improvement in the repeated inflammatory markers or imaging.

The fourth case was a 54-year-old male presented with a diagnosis of Brucella spondylodiskitis with a Brucella titer of 1:1280. He received three-drug therapy (streptomycin, ciprofloxacin, and doxycycline) for 6 months and then stopped as per the treating physician due to improvement in symptoms and imaging, but the Brucella titer was still high. After 3 months, symptoms returned, and the same high Brucella titer was observed. The treatment was resumed for 3 months until symptoms cleared, and the titer improved to 1:160.

In our study, we encountered one case of death within 90 days. The patient was a 73-year-old lady with a history of diabetes and hypertension. She was admitted as a case of urinary tract infection and received multiple antimicrobials. During admission, she was diagnosed with focal brucellosis based on a positive blood culture and MRI findings consistent with features of sacrolilitis and symphysitis. She completed the treatment of brucellosis for 3 months with a three-drug regimen (doxycycline, rifampin, and gentamicin). However, she died after one week after completing the 3 months duration of therapy. She had complicated hospital course with multiorgan failure, and her death was not likely attributed to brucellosis.

## Discussion

In this specified complicated brucellosis population with osteoarticular disease, results showed a minimum number (4.88%) of unfavorable outcomes, the majority of the cases (75.60%) in the study were on three-drug regimen, and 80.49% of them were treated for more than 3 months.

Osteoarticular disease is universally the most common complication of brucellosis, and three distinct forms exist: peripheral arthritis, sacroiliitis, and spondylitis [16].

The spinal column is one of the most frequently affected sites, with a highly variable incidence (2–54%) in different studies [10, 12, 13, 17–19]. Spondylodiscitis was shown to be the most common presentation in our study (46.34%).

Brucellosis with peripheral skeleton involvement is less prevalent compared with vertebral features [14], as arthritis occurs in 14–26% of the patients suffering from acute, sub-acute, or chronic brucellosis [20, 21]. Knee, hip, and ankle joints are among the most common peripheral regions affected by brucellosis, and these patients present with arthritis [15, 22]. Our study showed predominantly knee involvement in 15 (18.29%) of the total population. Twelve (80%) patients presented with septic knee arthritis. While the remaining 3 (20%) complained of knee pain and swelling only. Compared to the reported incidence in the literature (14–26%), the finding in our study falls in the same range, as it was 12 out of 82 (14.6%). Such an emphasis on the importance of including brucellosis as a differential diagnosis of knee arthritis is important in regions where it is prevalent.

Concerning the diagnosis, all patients in our cohort had a positive serology(1:320), which has been referenced as positive in endemic areas, but the rate of positive blood culture was 40.2%, which is also matching the literature, which ranges from 15 to 70% [23].

The relapse rate or unfavorable outcomes have been previously reported in spinal involvement between 0 and 26% using different regimen [24]. While our percentage is 4.88%, which is likely explained by the longer duration and greater number of medications used.

There is no consensus or strong evidence for the treatment of localized brucellosis related to the regimen to be used. Two of the larger pieces of evidence about spinal brucellosis found no significant therapeutic difference between different antibiotics [25, 26]. However, in a prospective study done by Gangi et al., a combination regimen of doxycycline plus rifampin and aminoglycoside showed a better response rate compared to others. Also, a similar result was found in a randomized trial by Bayindir et al. [24, 27]. Within the same context, our study reflected the lack of consensus in real life about the preferred regimen, but the predominance of 32 patients (39.02%) received the doxycycline + rifampicin +/- aminoglycoside regimen.

Despite the frequency of brucellosis in developing countries, including Middle Eastern countries, the optimal treatment regimen for the complicated disease is still controversial. Although the duration varies considerably in different studies, treatment with antibiotics administered for a period between 6 and 12 weeks sounds acceptable [24, 28, 29].

While some evidence may suggest that prolonging therapy is not required, others suggest that 6 weeks should be used only for non-complicated brucellosis disease. The need to prolong therapy beyond 3 months seems particularly important for complicated spinal involvement with any extension of infection through paravertebral and epidural spaces [25–27]. The practice in our hospital is to treat at least for 3 months; only 9 patients received treatment for the duration of 6 weeks or less. Of note, 3 out of 4 unfavorable outcomes were noticed for treatment durations less than 3 months.

Concerning the safety of the regimen, a better-designed study is required to address the safety of the different regimens, especially with localized brucellosis, as the duration of therapy is prolonged, and the side effects are expected to happen more frequently. Extrapolating from the data about the non-localized brucellosis, in the meta-analysis, the side effects of using different regimens showed a minimum number of serious side effects leading to the discontinuation of therapy, and the highest was 5% with the rifampicin and cotrimoxazole regimen [30]. In our study, serious side effects were noted to be secondary to ciprofloxacin in three patients.

We acknowledge that it is difficult to compare the regimens because of the very low relapse rate. Ultimately, the results of all statistical tests should be interpreted cautiously because of the limited sample size and very low rate of unfavorable outcomes.

On the same page, it would not be precise to conclude the best duration from our study, despite the fact that most of the unfavorable outcomes were seen in treatments that lasted less than 3 months. The overall findings in the study likely reflect the real-world practice of complicated brucellosis and the tendency to prolong the duration of treatment, which could be unnecessary. Another limitation is that the study is retrospective in nature, which makes judging the clinical outcome in a timely manner difficult, especially with some missing data. One of the strengths of our study is that it includes a homogenous population of complicated or localized diseases, assessing a comprehensive cure in addition to the radiological aspects of the treatment. Our evidence is an addition to the scarcity of new evidence of brucellosis osteoarticular disease in the region. Studies in the future will likely need to assess the duration of therapy better in an era where there is no consensus or strong evidence about how long is enough. What has been noticed is that the relapse rate is low in our cohort; thus, how we can avoid needless, longer therapy is the important question to be answered. Otherwise, such variability and longer therapy would continue.

## Conclusion

Safe and favorable outcomes have been noticed in our cohort of patients with complicated brucellosis treated mainly with a three-drug regimen and with a longer duration of therapy for complicated brucellosis with osteoarticular disease. Further studies will be important to determine the exact duration needed for the treatment without extending it unnecessarily.

## Supporting information

S1 Checklist Human participants research checklist. (DOCX)

S1 Data (XLSX)
